# Postpartum thromboembolism risk by CHA_2_DS_2_‐VASc score in patients with atrial fibrillation or flutter during pregnancy

**DOI:** 10.1002/pmf2.70082

**Published:** 2025-07-30

**Authors:** Virginia Y. Watkins, Sarah C. Snow, Miriam L. Estin, Cary C. Ward, Marie‐Louise Meng, Tracy Truong, Anna Xu, Jerome J. Federspiel

**Affiliations:** ^1^ Division of Maternal Fetal Medicine Department of Obstetrics and Gynecology Duke University School of Medicine Durham North Carolina USA; ^2^ Division of Cardiology Department of Medicine Duke University School of Medicine Durham North Carolina USA; ^3^ Division of Women's Anesthesia Department of Anesthesiology Duke University School of Medicine Durham North Carolina USA; ^4^ Department of Biostatistics and Bioinformatics Duke University School of Medicine Durham North Carolina USA; ^5^ Department of Population Health Sciences Duke University School of Medicine Durham North Carolina USA; ^6^ Division of Hematology Department of Medicine Duke University School of Medicine Durham North Carolina USA

**Keywords:** anticoagulation, atrial fibrillation, flutter, stroke, thromboembolism

## Abstract

**Introduction:**

To evaluate whether rates of postpartum thromboembolism (TE), transient ischemic attack (TIA), and cerebrovascular accident (CVA) among patients with a history of AF/AFL differed based on CHA_2_DS_2_‐VASc scores 1 versus ≥ 2 (i.e., those with risk factors beyond female sex).

**Methods:**

This retrospective cohort study used the 2016–2021 National Readmissions Database to identify patients with a diagnosis of atrial fibrillation (AF)/atrial flutter (AFL) at delivery. The primary outcome was rate of acute thromboembolic events (TE/TIA/CVA) during delivery hospitalization or within 42 days following discharge. Secondary outcomes included cerebrovascular morbidity specifically (TIA/CVA), severe maternal morbidity (SMM), non‐transfusion and cardiovascular‐related SMM, mode of delivery, preterm birth, and patient disposition. CHA_2_DS_2_‐VASc scores were calculated for each patient at the index hospitalization and scores compared as 1 and ≥ 2. After adjusting for facility characteristics and obesity, a modified Poisson regression model was used to estimate relationships between groups.

**Results:**

The cohort consisted of 3574 delivery hospitalizations with AF/AFL, corresponding to a national estimate of 6816. Of those, 5178 (76.0%) had a CHA_2_DS_2_‐VASc score of 1 and 1638 (24.0%) had a CHA_2_DS_2_‐VASc ≥ 2. After adjusting for facility and patient characteristics, patients with a CHA_2_DS_2_‐VASc ≥ 2 were more likely to experience an acute thromboembolic event (3.9% vs. 1.5%, aRR 2.04 [1.16, 3.60]), SMM (23.5% vs. 11.4%, aRR 1.77 [1.47, 2.14]), and pulmonary edema or acute heart failure (11.4% vs. 1.3%, aRR 7.12 [4.46, 11.01]) compared with those with CHA_2_DS_2_‐VASc of 1 over the 6‐week postpartum period. Patients with AF/AFL and CHA_2_DS_2_‐VASc of 1 had a TIA/CVA rate of 1.4%, while those with CHA_2_DS_2_‐VASc ≥ 2 had a TIA/CVA rate of 2.5%, with no statistically significant difference between groups in adjusted models (aRR 1.37 [0.74, 2.52]). No significant association was observed between CHA_2_DS_2_‐VASc score and need for cardioversion or onset of hemodynamic shock.

**Conclusions:**

Pregnant patients with a history of AF/AFL and a CHA_2_DS_2_‐VASc score ≥ 2 are significantly more likely to experience an acute thromboembolic event following delivery than those with a CHA_2_DS_2_‐VASc of 1. Additionally, baseline rates of TIA/CVA for pregnant individuals with AF/AFL but no additional risk factors beyond sex were 1.4% during the 6‐week postpartum period, suggesting that pregnancy and postpartum states have increased risk not accounted for by the CHA_2_DS_2_‐VASc score. Prospective studies are needed to better understand risk for pregnant and postpartum patients with AF/AFL and to determine optimal management strategies.

## 1 INTRODUCTION

Cardiovascular disease is one of the most prevalent comorbidities during pregnancy, affecting 1%–4% of all pregnancies and accounting for approximately 25% of maternal morbidity and mortality [1]. Cardiac arrhythmias are one of the most common cardiac complications and can present in patients with and without a history of heart disease secondary to the physiologic cardiovascular and hemodynamic changes of pregnancy [2, 3, 4, 5]. Atrial fibrillation (AF) and atrial flutter (AFL) are the most common newly diagnosed sustained arrhythmia during pregnancy and can be associated with maternal hemodynamic instability requiring urgent intervention, as well as multiple obstetrical and fetal complications [6, 7, 8].

In nonpregnant individuals, the CHA_2_DS_2_VASc score is used to guide which patients with nonvalvular AF/AFL are candidates for anticoagulation, with anticoagulation typically recommended for individuals with scores ≥ 2. The CHA_2_DS_2_VASc score assigns one point for female sex, thus requiring only one additional risk factor to be present in pregnant populations to be considered intermediate risk. Notably, there is no pregnancy‐specific risk stratification score; it has therefore been recommended that the anticoagulation plan during pregnancy be created considering a patient's CHA_2_DS_2_VASc score, similarly to nonpregnant individuals [8, 9, 10, 11, 12]. However, a small study by Lindley et al. found that the CHA_2_DS_2_VASc score underestimated thrombosis risk during pregnancy with systemic thromboembolism occurring in 2/35 (5.7%) patients with a CHA_2_DS_2_VASc score of only 1 and 1/13 (7.7%) of patients with a CHA_2_DS_2_VASc score of 2 [13]. Additionally, there are limited data regarding the usage or utility of CHA_2_DS_2_VASc scores in the postpartum period.

AF/AFL is associated with arterial (systemic) thromboembolism due to clots forming in the heart and then traveling into the systemic circulation, with CVA/TIA being the most common manifestation. However, data suggest that AF/AFL is also associated with increased risk of venous thromboembolism (VTE), which is far more common than stroke in obstetric populations [14, 15, 16]. A recent large meta‐analysis showed that AF was associated with increased risk of VTE, with the highest risk in the first 3–6 months after AF diagnosis [16]. The pathophysiologic relationship between AF/AFL and VTE remains unclear, but some suggest that it may be shared underlying risk factors such as diabetes, hypertension, heart failure, rates of hospitalization, and other factors that may account for this association [16]. Given that pregnancy is a thrombogenic state, pregnancy may confer additional risk in this patient population.

The goal of this study is to evaluate rates of thromboembolic events, namely thromboembolism (TE) (systemic and venous), transient ischemic attack (TIA), and cerebrovascular accident (CVA), after delivery in patients with a history of AF/AFL stratified by CHA_2_DS_2_VASc score of 1 and ≥ 2 at the time of delivery.

## 2 MATERIALS AND METHODS

This retrospective cohort study used the Nationwide Readmissions Database (NRD) to identify patients with an inpatient diagnosis code of AF/AFL at the index delivery hospitalization. The NRD is an all‐payer administrative dataset that captured approximately 60.8% of inpatient admissions from non‐Federal hospitals in the United States in 2021 [16]. It is a nationally representative, stratified sample of hospital readmissions from US community hospitals, excluding rehabilitation, long‐term acute care, and federal facilities. The NRD allows the estimation of national readmission rates using discharge weights (DISCWT), hospital clusters (HOSP_NRD), and hospital‐level stratification (NRD_STRATUM) by region, location, teaching status, bed size, and ownership. To ensure sufficient representation, strata were collapsed as needed—first across ownership/control, then location, and finally bed size—while preserving region and teaching status. The NRD includes patient demographics such as age and primary payer, along with *International Classifications of Disease, 10^th^ edition* (ICD‐10) diagnosis and procedure codes to identify clinical conditions, including those present at delivery hospitalization. The NRD cannot capture information regarding inpatient or outpatient prescriptions. Subsequent readmissions up to 6 weeks from the index hospitalization were queried to assess for primary and secondary outcomes. This study was deemed exempt from review by the Duke University Health System Institutional Review Board as the NRD is a Limited Data Set as defined by the Health Insurance Portability and Accountability Act.

We used a validated algorithm updated for ICD‐10 coding to identify delivery hospitalizations [17]. Patients with a confirmed primary or secondary diagnosis code of AF/AFL between 2016 and 2021 were included, with ICD‐10 code I48.* used to identify our cohort. CHA_2_DS_2_VASc scores were calculated for each patient at the index hospitalization and patients were grouped as scores of 1 and ≥ 2 using ICD‐10 diagnosis codes for CHA_2_DS_2_VASc score components: congestive heart failure, chronic hypertension, diabetes, history of TE/TIA/CVA, and vascular disease (Table S1). The cut‐off of at least 2 points was chosen as this is the standard cut‐off generally used in nonpregnant individuals for determining candidacy for anticoagulation. As expert consensus recommends anticoagulation in patients with AF/AFL during pregnancy and postpartum be managed similarly to nonpregnant individuals, it is logical to use the same cut‐off [8, 12].

The primary outcome was acute thromboembolic event (TE/TIA/CVA) from delivery hospitalization until 6 weeks’ postpartum. Secondary outcomes included TIA/CVA for cerebrovascular morbidity alone, severe maternal morbidity (SMM), a composite event as defined by the Center for Disease Control and Prevention (CDC), non‐transfusion SMM and cardiovascular‐related SMM (acute myocardial infarction, aortic aneurysm, cardiac arrest or ventricular fibrillation, conversion of cardiac rhythm, heart failure or cardiac arrest during surgery or procedure, pulmonary edema or acute heart failure, puerperal cerebrovascular disorders, and shock), mode of delivery, preterm birth, and patient disposition [18]. ICD‐10 codes for acute TE (systemic and venous), TIA, and CVA as well as individual outcomes included in the CDC definition of SMM were included in the model (Table S2).

Annual percentage of patients delivering with an AF/AFL diagnosis during pregnancy and rates of acute thromboembolic event between 2016 and 2021 were calculated. Baseline patient characteristics were compared between patients with a CHA_2_DS_2_VASc of 1 and ≥ 2 on index delivery hospitalization. Continuous outcomes were compared using a weighted linear regression model and are reported as mean (standard deviation), and binary/categorical variables were compared using a weighted χ^2^ test and are reported as frequency (percentage). The association between CHA_2_DS_2_VASc groups and primary and secondary outcomes were assessed using a modified Poisson regression model, incorporating DISCWT, hospital clusters, and hospital stratification provided by NRD, with and without covariates [19, 20]. Covariates included are age, median household income for patient's ZIP code, primary payer, hospital size, hospital location and teaching status, and obesity in pregnancy. Adjusted risk ratios (aRRs) and 95% confidence intervals (CIs) are reported. Regression analysis was not performed for acute VTE, acute myocardial infarction, aneurysm, cardiac arrest or ventricular fibrillation, and puerperal cerebrovascular disorders due to low event rates and vendor limitations with reporting.

A separate analysis was performed excluding deliveries that occurred during the COVID‐19 pandemic and included only patients delivering with an AF/ALF diagnosis during pregnancy between 2016 and February 2020. This subgroup analysis was performed given the increased maternal morbidity and mortality associated with the pandemic as well as the known thrombogenicity of the COVID‐19 infection. Additionally, we assessed effect modification by mode of delivery in a model with the interaction term for CHA_2_DS_2_VASc groups and mode of delivery for the primary and secondary outcomes. This analysis was performed as cesarean delivery confers an elevated risk of postpartum thromboembolism and is often considered when determining candidacy for postpartum thromboprophylaxis [21]. All analyses were conducted in SAS 9.4 (SAS Institute Inc.) and R 4.4.0 (R Core Team, 2024).

## 3 RESULTS

**FIGURE 1:**
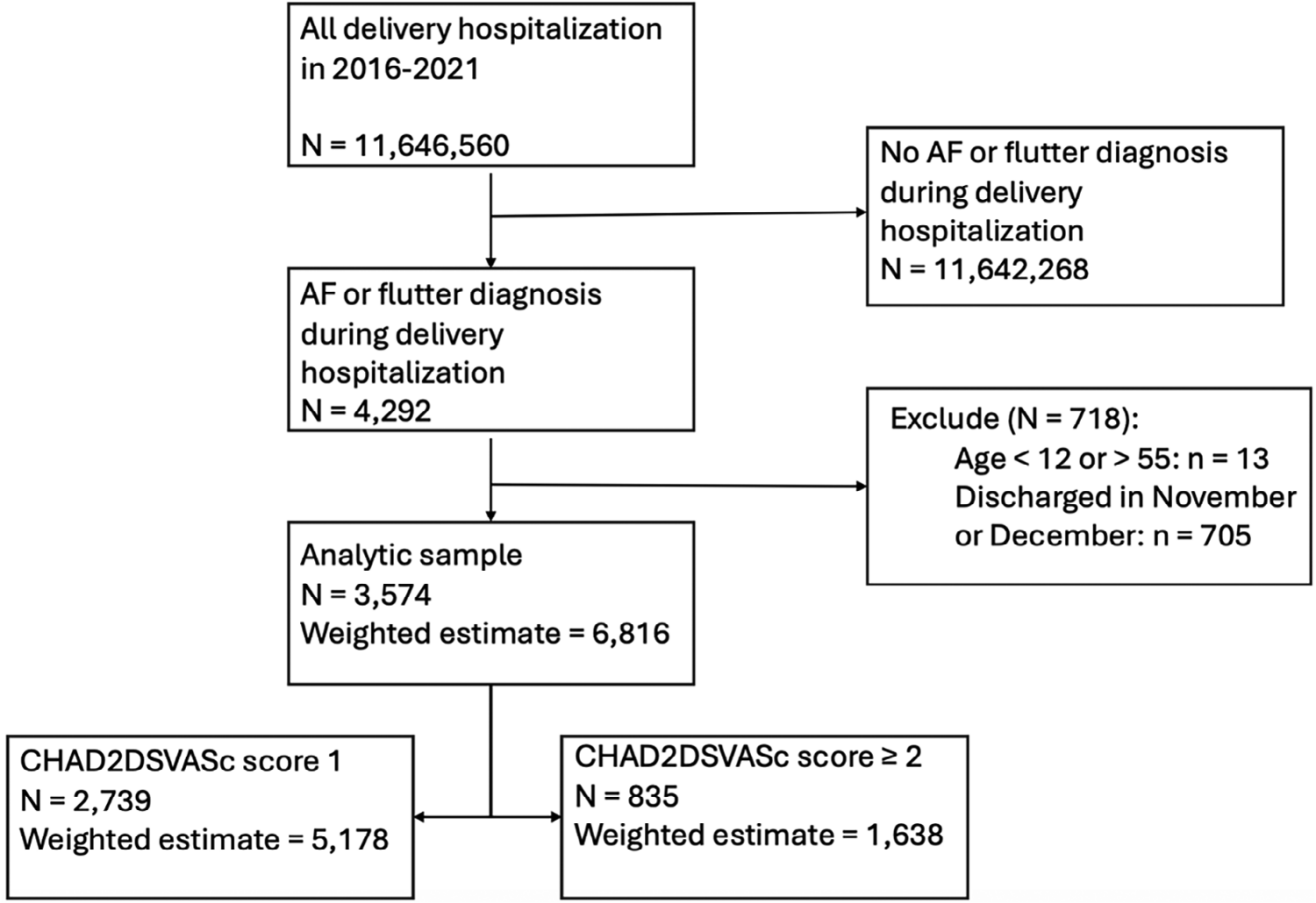
Derivation of study cohort.

We identified a cohort of 3574 patients with an ICD‐10 diagnosis code of AF/AFL at delivery hospitalization between January 2016 and October 2021, corresponding to a national estimate of 6816 (Figure 1). Results are presented as weighted national estimates. Among the overall cohort, 5178 (76.0%) had a CHA_2_DS_2_VASc score of 1 and 1638 (24.0%) had a CHA_2_DS_2_VASc score ≥ 2. Atrial fibrillation and flutter diagnosis rate at delivery hospitalization increased from 2016 (3.3 per 10,000) to 2021 (4.6 per 10,000).

Patients with a CHA_2_DS_2_VASc ≥ 2 were older, more like to reside in an area with lower median household incomes, more likely to be insured by Medicaid or Medicare, and were more likely to deliver at a metropolitan teaching facility compared to patients with a CHA_2_DS_2_VASc of 1 (Table 1). Patients with a CHA_2_DS_2_VASc ≥ 2 were more likely to have preexisting comorbid conditions including asthma, cardiac valve disease, ischemic heart disease, chronic kidney disease, obesity, and tobacco use disorder compared with patients with a CHA_2_DS_2_VASc of 1 (Table 1). There were no differences in the following comorbid conditions between groups: alcohol abuse, congenital heart disease, drug abuse, sickle cell disease, or systemic lupus erythematous (Table 1). The most common comorbidity that placed patients into the CHA_2_DS_2_VASc ≥ 2 group was chronic hypertension (*n* = 1105 (67.5%)).

**TABLE 1:** Baseline demographic characteristics.

	CHA_2_DS_2_VASc 1 (*N* = 5178)	CHA_2_DS_2_VASc ≥ 2 (*N* = 1638)	*p* value
Patient age (years), mean (SD)	30.8 (0.1)	33.4 (0.2)	< 0.001
Median household income for patient's ZIP code			< 0.001
Quartile 1 (lowest)	1464 (28.5)	562 (34.7)	
Quartile 2	1313 (25.6)	471 (29.1)	
Quartile 3	1300 (25.3)	340 (21.0)	
Quartile 4 (highest)	1057 (20.6)	246 (15.2)	
Primary payer			0.001
Medicaid	2185 (42.3)	776 (47.3)	
Medicare	99 (1.9)	103 (6.3)	
Private	2705 (52.3)	707 (43.2)	
Self‐Pay	49 (0.9)	21 (1.3)	
Other	133 (2.6)	31 (1.9)	
Hospital size			< 0.001
Small	639 (12.3)	161 (9.8)	
Medium	1214 (23.4)	304 (18.5)	
Large	3325 (64.2)	1173 (71.6)	
Hospital location and teaching status			0.018
Metropolitan, non‐teaching	793 (15.3)	174 (10.6)	
Metropolitan, teaching	4089 (79.0)	1384 (84.5)	
Non‐metropolitan	296 (5.7)	79 (4.8)	
Comorbid conditions			
Alcohol abuse	23 (0.4)	^a^	0.609
Asthma	555 (10.7)	285 (17.4)	< 0.001
Cardiac valve disease	258 (5.0)	130 (8.0)	0.001
Congestive heart failure	22 (0.4)	138 (8.5)	< 0.001
Ischemic heart disease	15 (0.3)	125 (7.6)	< 0.001
Chronic kidney disease	15 (0.3)	46 (2.8)	< 0.001
Congenital heart disease	175 (3.4)	42 (2.5)	0.201
Drug abuse	249 (4.8)	100 (6.1)	0.134
Obesity in pregnancy	1116 (21.6)	710 (43.3)	< 0.001
Sickle cell disease	16 (0.3)	^a^	0.224
Systemic lupus erythematosus	34 (0.7)	23 (1.4)	0.056
Tobacco use disorder	374 (7.2)	160 (9.8)	0.024

*Note*: Data represent *n* (%) unless otherwise denoted.

^a^ *N* ≤ 10; value suppressed by data vendor limitations.

**FIGURE 2:**
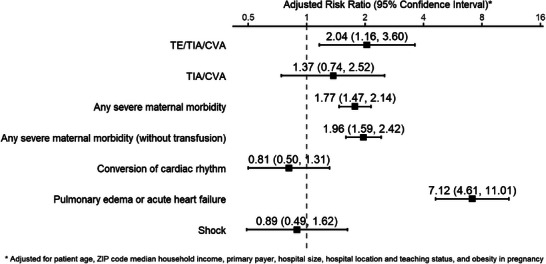
Covariate‐adjusted primary and secondary outcomes.

After adjusting for facility and patient characteristics, patients with a CHA_2_DS_2_VASc ≥ 2 were significantly more likely to experience an acute thromboembolic event (3.9% vs. 1.5%, aRR 2.04 [1.16, 3.60]), SMM (23.5% vs. 11.4%, aRR 1.77 [1.47, 2.14]), non‐transfusion SMM (21.5% vs. 9.4%, aRR 1.96 [1.59, 2.42]) and pulmonary edema or acute heart failure (11.4% vs. 1.3%, aRR 7.12 [4.61, 11.01]) (Figure 2) over the 6‐week postpartum period. When evaluating cerebrovascular events, patients with AF/AFL and CHA_2_DS_2_VASc of 1 had a TIA/CVA rate of 1.4%, while those with CHA_2_DS_2_VASc ≥ 2 had a TIA/CVA rate of 2.5% (Table 2). There was no significant difference in TIA/CVA rates between groups in adjusted models (aRR 1.37 [0.74, 2.52]) (Figure 2). There were higher rates of puerperal cerebrovascular disorders (1.4% vs. 0.3%, *p* < 0.001) in patients with a CHA_2_DS_2_VASc ≥ 2, although modified Poisson model was not fit for this outcome given overall low event rates. There was no difference in rates of conversion of cardiac rhythm (3.4% vs. 3.8%, aRR 0.81 [0.50, 1.31]) or shock (1.9% vs. 2.0%, aRR 0.89 [0.49, 1.62]) (Figure 2).

**TABLE 2:** Primary and secondary outcomes.

	CHA_2_DS_2_VASc 1 (*N* = 5178)	CHA_2_DS_2_VASc ≥ 2 (*N* = 1638)	*p* value
Delivery type			< 0.001
Cesarean delivery	2582 (50.4)	1026 (63.6)	
Operative vaginal	194 (3.8)	70 (4.4)	
Spontaneous vaginal	2345 (45.8)	517 (32.0)	
Preterm birth	1014 (19.6)	569 (34.7)	< 0.001
Discharge disposition of patient			< 0.001
Against medical advice	11 (0.2)	15 (0.9)	
Alive, destination unknown	^a^	^a^	
Died	30 (0.6)	^a^	
Home health care	100 (1.9)	70 (4.3)	
Other transfer	11 (0.2)	^a^	
Routine	5010 (96.8)	1533 (93.6)	
Transfer to short‐term hospital	16 (0.3)	^a^	
Acute TE/TIA/CVA^b^	78 (1.5)	64 (3.9)	< 0.001
Acute TE	^a^	23 (1.4)	< 0.001
Acute TIA/CVA	71 (1.4)	41 (2.5)	0.033
Any Severe Maternal Morbidity (include blood transfusion)^b^	592 (11.4)	385 (23.5)	< 0.001
Any Severe Maternal Morbidity (exclude blood transfusion)^b^	489 (9.4)	353 (21.5)	< 0.001
Cardiovascular‐related Severe Maternal Morbidity			
Acute myocardial infarction^b^	24 (0.5)	^a^	0.555
Aneurysm^b^	^a^	^a^	0.031
Cardiac arrest or ventricular fibrillation^b^	32 (0.6)	14 (0.9)	0.450
Cardioversion^b^	196 (3.8)	56 (3.4)	0.594
Heart failure/arrest during surgery or procedure^b^	^a^	^a^	0.226
Pulmonary edema or acute heart failure^b^	70 (1.3)	186 (11.4)	< 0.001
Puerperal cerebrovascular disorders^b^	16 (0.3)	23 (1.4)	0.001
Shock^b^	104 (2.0)	32 (1.9)	0.905

*Note*: Data represent *n* (%).

^a^ *N* ≤ 10; value suppressed by data vendor limitations.

^b^ Morbidity condition recorded up to 6 weeks from index hospitalization.

Patients with a CHA_2_DS_2_VASc ≥ 2 were significantly more likely to have a cesarean delivery (63.6% vs. 50.4%, *p* < 0.001), preterm birth (34.7% vs. 19.6%, *p* < 0.001), and require home health care at hospital discharge (4.3% vs. 1.9%, *p* < 0.001) compared with those with a CHA_2_DS_2_VASc of 1 (Table 2).

In the sensitivity analysis where we included only deliveries from January 2016 through February 2020 in order to exclude those occurring during the COVID‐19 pandemic, the analytic dataset consisted of 2415 deliveries corresponding to a weighted national estimate of 4590. Among the 4590 deliveries, 3539 had a CHA_2_DS_2_VASc score of 1 and 1050 had a CHA_2_DS_2_VASc ≥ 2. After adjusting for confounding factors, patients with a CHA_2_DS_2_VASc ≥ 2 during this time period had a significantly higher rate of acute thromboembolic event compared those with a CHA_2_DS_2_VASc of 1 (3.7% vs. 1.4%, aRR 2.62 [1.38, 4.98]).

When we evaluated outcomes by mode of delivery, we found that the association between CHA_2_DS_2_VASc ≥ 2 and each outcome did not vary by mode of delivery (Table 3). The primary outcome, acute thromboembolic event, had a borderline statistical significance (*p* = 0.061) and it was still meaningful to show the directionality of the interaction. We observed that among vaginal deliveries, CHA_2_DS_2_VASc ≥2 was associated with a greater risk of acute thromboembolic event compared to those with CHA_2_DS_2_VASc of 1 (aRR 4.83 [1.57, 14.97]). Conversely, there was no statistically significant association between CHA_2_DS_2_VASc ≥ 2 and acute thromboembolic event among cesarean deliveries (aRR 1.48 [0.81, 2.70]).

**TABLE 3:** Association between CHA_2_DS_2_VASc ≥ 2 and clinical outcomes by mode of delivery.

Outcomes	Interaction *p* values
Acute TE/TIA/CVA	0.061
Acute TIA/CVA	0.619
Any severe maternal morbidity (include blood transfusion)	0.300
Any severe maternal morbidity (exclude blood transfusion)	0.110
Conversion of cardiac rhythm	0.979
Pulmonary edema or acute heart failure	0.494
Shock	0.306

## 4 DISCUSSION

In this retrospective cohort study, we found that rates of AF/AFL affecting pregnancy are increasing. Pregnant patients with a history of AF/AFL and a CHA_2_DS_2_VASc score ≥ 2 (i.e., with risk factors beyond female sex) were significantly more likely to experience an acute thromboembolic event, SMM, and pulmonary edema or acute heart failure during the postpartum period than those with a CHA_2_DS_2_VASc score of 1. Patients with AF/AFL had higher rates of TIA/CVA than previously established estimates in non‐pregnant individuals. The increased risk of acute thromboembolic event in patients with a CHA_2_DS_2_VASc > 2 remained significant when excluding patients who delivered during the COVID‐19 pandemic.

Pregnancy‐related morbidity has increased over the past three decades, with the CDC's Pregnancy Mortality Surveillance estimating approximately 10 deaths per 100,000 live births in the early 1990s and approximately 17 deaths per 100,000 live births in the years preceding COVID‐19 [18]. In an observational survey of pregnancy‐related deaths from 2011 to 2013, Creanga et al. found that cardiovascular‐specific conditions accounted for approximately 25% of these deaths, with thromboembolism claiming approximately 10%–15% [22, 23]. Fortunately, current data suggests that adverse maternal cardiac events are rare in patients with AF/AFL during pregnancy. A recent population‐based study by Lee et al. at Kaiser Permanente Southern California hospitals between 2003 and 2013 evaluated 157 pregnancies complicated by AF/AFL and found that only 2 patients developed heart failure with no cerebrovascular accidents, embolic events, or cases of maternal death [24]. Moreover, only 5 patients in this study received anticoagulation. However, this study only evaluated adverse cardiac events *during* pregnancy and clinically significant arrhythmias postpartum, failing to capture a range of adverse outcomes that may be most likely to occur in the postpartum period.

The treatment of AF/AFL during pregnancy and postpartum poses unique challenges. In general, management is similar to that of non‐pregnant individuals. The mainstay of therapy is rate control with a beta blocker, calcium channel blocker, and/or digoxin, with cardioversion considered safe during pregnancy in refractory cases or hemodynamic instability [8, 25, 26, 27, 28, 29, 30, 31, 32]. When antiarrhythmic agents are needed, flecainide or sotalol may be used [33]. Recommendations regarding anticoagulation in patients with AF/AFL are largely based on case reports and expert opinion. The Heart Rhythm Society (HRS) released guidelines for the management of arrhythmias during pregnancy in 2023 [8]. Currently, the HRS recommendations on anticoagulation in these cases note that in “pregnant patients with AF or AFL and additional risk factors that place them at high risk for thromboembolism, anticoagulation is recommended as in the nonpregnant patient” [8]. If anticoagulation is recommended, heparins or, in selected cases, warfarin are used; direct oral anticoagulants are reserved for postpartum patients who are not breastfeeding [34].

Our results showed that in patients who had a vaginal delivery, the primary outcome of thromboembolic event rates was significantly higher in patients with a CHA_2_DS_2_VASc ≥ 2 compared to those with CHA_2_DS_2_VASc score of 1 and it was not statistically significant in patients who had cesarean deliveries. One hypothesis for this finding would be higher rates of prophylactic anticoagulation prescriptions in patients undergoing cesarean, although this cannot be confirmed in our cohort. The high rates of our primary outcome among both vaginal and cesarean deliveries underscore the importance of consideration of anticoagulation in patients with a history of AF/AFL, regardless of mode of delivery.

There is currently limited information on the utility of CHA_2_DS_2_VASc scores during pregnancy and postpartum, yet current guidelines rely on CHA_2_DS_2_VASc score. Pregnancy is not a risk factor in the CHA_2_DS_2_VASc score, but the pregnant and postpartum states are undeniably thrombogenic. As part of a physiologic adaptation of pregnancy, fibrinogen doubles, clotting factors I, II, VII, VIII, IX, and XII increase 20%–1000%, free protein S drops 40%–60%, and compression of the inferior vena cava and pelvic veins by the gravid uterus results in venous stasis [35, 36]. In a retrospective cohort study of 53 cases, Lindley et al. identified 2 cases of systemic thromboembolism out of 35 (5.7%) in patients with CHA_2_DS_2_VASc score of 1 and 1 case (7.7%) in patients with CHA_2_DS_2_VASc of 2 [13]. In our study, patients with a CHA_2_DS_2_VASc score of 1 had a thromboembolic event rate of 1.5% over a 6‐week period. This was primarily driven by acute TIA/CVA, which had an event rate of 1.4%. This important finding shows that pregnant individuals with AF/AFL are at higher risk for TIA/CVA than previously established rates (0.6% per year for TIA/CVA, 0.9% per year for TE/TIA/CVA), for non‐pregnant individuals with a score of 1 [37]. This finding underscores the importance of additional studies evaluating the validity of CHA_2_DS_2_VASc in pregnancy and postpartum. Our study also suggests that with a baseline risk of 1.4% of TIA/CVA over a 6‐week period, prospective studies are needed in this patient population to better estimate risk and establish algorithms to determine who would benefit from anticoagulation.

The NRD does not contain information regarding anticoagulation prescription among the cohort individuals. We are therefore unable to determine which patients in our cohort may have received aspirin, prophylactic or therapeutic anticoagulation during their pregnancies and in the postpartum period; we are therefore unable to comment on the recommended dosage of anticoagulation for this patient population. The authors acknowledge this as a significant limitation to our study, and that further work employing datasets with these elements is an area where urgent investigation is needed. The lack of large scale, clinically granular obstetrics data in the United States is a common limitation for rare disease research in pregnancy; for this reason, we have previously proposed the creation of national EMR‐augmented obstetrics registry [38].

However, although this limitation does not allow recommendations regarding postpartum anticoagulation in patients with AF/AFL, the preliminary data deserve consideration. While guidelines for postpartum thromboprophylaxis candidacy vary by society, the American Society of Hematology conducted a multidisciplinary panel in which they polled experts regarding risk threshold for recommending postpartum thromboprophylaxis. Panel responses ranged from 1% to 3%, with the majority of experts recommending chemical thromboprophylaxis if risk reaches 1% [39]. With this threshold, based on our study findings, patients with a history of AF/AFL would be considered for at least thromboprophylaxis irrespective of CHA_2_DS_2_VASc score. Further studies are needed to clarify the role of prophylactic versus therapeutic anticoagulation doses in this patient population. At this time, given the lack of data, the risks and benefits of anticoagulation should be weighed on an individual basis for pregnant or postpartum patients with AF/AFL.

There are several strengths of our study. In utilizing the NRD, we can use weighted national estimates to examine an overall rare and poorly studied diagnosis in our pregnant population, AF/AFL. Our ability to stratify groups by CHA_2_DS_2_VASc scores allows close examination of this tool in pregnant individuals. The NRD allowed us to examine rare outcomes including thromboembolic event rates. As the NRD is a large, representative dataset that reflects all payors, the design of this study allows generalizability to the general population. Our study is novel in that it examines *postpartum* outcomes in patients with a history of AF/AFL and compares outcomes based on CHA_2_DS_2_VASc score. This finding provides more data to a largely controversial area of postpartum thromboprophylaxis, let alone full‐dose anticoagulation candidacy.

There are limitations to the study that should be considered. Our study utilized ICD‐10 codes assigned at the time of discharge through the NRD, which may result in overestimation of outcomes. A recent study highlighted the limitations in accurate SMM reporting when using delivery claims, noting a false positive discovery rate of 19.0% [40]. The development of computable phenotypes for true de novo thromboembolism in delivery claims and/or access to national obstetrical registries would help strengthen our study and validate our findings. We are unable to account for patients with several pregnancies and delivery hospitalizations greater than one year apart in our cohort, although we did exclude patients with subsequent deliveries in the same year. Additionally, we are also unable to comment on the use of rate control medications or determine the extent of the AF/AFL diagnosis, as we suspect that more refractory cases of tachyarrhythmias may result in higher TE rates. We are also unable to determine the timing of the diagnosis of the arrhythmia nor conversion back to normal sinus rhythm and its proximity to delivery hospitalization. Finally, while we adjusted for facility characteristics in our analysis, we are unable to account for variation in hospital practice. This represents a potential confounder in our analysis given the broad range of prescribing practices for postpartum thromboprophylaxis.

## 5 CONCLUSION

In conclusion, pregnant individuals with a history of AF/AFL and CHA_2_DS_2_VASc ≥ 2 have higher rates of postpartum thromboembolic event, overall and non‐transfusion SMM, and pulmonary edema or acute heart failure compared with patients with a history of AF/AFL and CHA_2_DS_2_VASc score of 1. Further, the baseline risk of TIA/CVA for pregnant patients with AF/AFL is high at 1.4% over the 6‐week postpartum period, suggesting that a closer look at postpartum thromboembolic risk levels and the role of anticoagulation in this patient population is needed. Prospective studies are necessary to better evaluate risk in this patient population as well as the role of prophylactic versus therapeutic anticoagulation dosage.

## CONFLICT OF INTEREST STATEMENT

The authors declare no conflicts of interest.

## Data Availability

The data that support the findings of this study are available from HEALTHCARE COST & UTILIZATION PROJECT. Restrictions apply to the availability of these data, which were used under license for this study.
